# Draft Genome Sequence of the Model Naphthalene-Utilizing Organism *Pseudomonas putida* OUS82

**DOI:** 10.1128/genomeA.01161-13

**Published:** 2014-01-16

**Authors:** Martin Tay, Dan Roizman, Yehuda Cohen, Tim Tolker-Nielsen, Michael Givskov, Liang Yang

**Affiliations:** Singapore Centre on Environmental Life Sciences Engineering (SCELSE), Nanyang Technological University, Singaporea; Costerton Biofilm Center, Department of International Health, Immunology and Microbiology, University of Copenhagen, Copenhagen, Denmarkb; School of Biological Sciences, Nanyang Technological University, Singaporec

## Abstract

*Pseudomonas putida* OUS82 was isolated from petrol- and oil-contaminated soil in 1992, and ever since, it has been used as a model organism to study the microbial assimilation of naphthalene and phenanthrene. Here, we report the 6.7-Mb draft genome sequence of *P. putida* OUS82 and analyze its featured pathways for biodegradation.

## GENOME ANNOUNCEMENT

*Pseudomonas putida* has served as a model organism for the degradation of complex carbon compounds, such as phenanthrene and naphthalene (1). Strains of *P. putida* have also been the organisms of choice for environmental and biological applications due to their nonpathogenic nature, unlike their close relative *Pseudomonas aeruginosa* (2), as well as their capacity to form biofilms (3)

Here, we present the draft genome of a well-characterized biodegradation strain, *P. putida* OUS82. *P. putida* OUS82 was isolated from oil-contaminated soil in 1992 (4), and ever since, it has been used as a model organism to study the microbial assimilation of naphthalene and phenanthrene (5). The draft genome of *P. putida* OUS82 consists of 164 contigs covering the length of 6,692,066 bp. Genome sequencing was performed on the Illumina MiSeq (250 bp per read). After quality trimming using the CLC Genomics Workbench version 6.5, 1,250,880 reads of good quality were assembled into contigs with an approximate coverage of 30× and a G+C content of 61.7%, with the largest contig being 528,016 bp in size. The draft genome of *P. putida* OUS82 contains the upper pathway for the degradation of phenanthrene and naphthalene to salicylate encoded by the previously reported 14-kb *pah* operon carried by a plasmid (6). The draft genome of *P. putida* OUS82 (contig 26) also contains the *sal* operon encoding the lower pathway for the degradation of phenanthrene and naphthalene. The *sal* operon contains 11 genes responsible for the degradation of salicylate and other intermediates into the tricarboxylic acid (TCA) cycle (7).

Open reading frames called from the genome using GeneMarkS were compared against proteins in nine other strains of *P. putida* (GenBank accession no. NC_017530.1, AF302763.1, CP000712.1, NC_010322.1, NC_019905.1, NC_002947.3, NC_017986.1, NC_015733.1, and NC_010501.1). Several of the unique open reading frames found in the OUS82 strain were found to be mapped to the degradation pathways of xylene, aminobenzoate, dioxin, bisphenol, chloroalkane, and chloroalkene within the KEGG orthology database. Based on the progressive alignment conducted on Mauve, *P. putida* OUS82 appears to be most closely related to the *P. putida* GB-1 strain (accession no. NC_010322.1).

### Nucleotide sequence accession number.

The complete coding sequence of *P. putida* OUS82 has been deposited in GenBank under the accession no. AZBL00000000.
